# Etiopathogenetic Mechanisms of Pulmonary Hypertension in Sleep-Related Breathing Disorders

**DOI:** 10.1155/2012/273591

**Published:** 2012-07-11

**Authors:** Ayodeji Adegunsoye, Siva Ramachandran

**Affiliations:** ^1^Department of Internal Medicine, Mercy Fitzgerald Hospital, Darby, PA 19018, USA; ^2^Sleep Medicine Services, Bryn Mawr Hospital, PA 19010, USA

## Abstract

Obstructive sleep apnea syndrome is a common disorder with significant health consequences and is on the rise in consonance with the obesity pandemic. In view of the association between sleep-disordered breathing and pulmonary hypertension as depicted by multiple studies, current clinical practice guidelines categorize obstructive sleep apnea as a risk factor for pulmonary hypertension and recommend an assessment for sleep disordered breathing in evaluating patients with pulmonary hypertension. The dysregulatory mechanisms associated with hypoxemic episodes observed in sleep related breathing disorders contribute to the onset of pulmonary hypertension and identification of these potentially treatable factors might help in the reduction of overall cardiovascular mortality.

## 1. Introduction

In consonance with the obesity pandemic, there is an increasing awareness of sleep-related breathing disorders (SRBDs) as a potentially treatable factor in reducing overall cardiovascular mortality. The spectrum of SRBD ranges from habitual snoring to obstructive sleep apnea (OSA) and increasing evidence shows that improved cardiovascular function may be obtained by early recognition and treatment of these disorders [1].

Over the past three decades, the pathophysiology of sleep-related breathing disorders (SRBDs) has been better understood and though the exact contributory pathways are still not clearly defined several studies allude to multi-factorial mechanisms being involved in the development of pulmonary hypertension in relation to SRBD [1].

Sleep apnea occurs in about 12 million US adults in their 4th to 6th decades of life and about a quarter of all those are over the age of 65 yrs. Nearly half of all nursing home residents have sleep apnea and 38,000 deaths annually are directly attributed to SRBD. With the prevalence of SRBD currently exceeding that of asthma in adults, the cardiovascular consequences of its associated comorbidities especially pulmonary hypertension (PH) have been of significant interest in recent years [1].

The most recent classification system of pulmonary hypertension was published in the 2009 European Society of Cardiology Guidelines where the definition of PH was based on an increased mean pulmonary arterial pressure >−25 mmHg at rest. This broadly encompasses all clinical subgroups of PH as outlined by the 4th World Symposium on Pulmonary Hypertension in Dana Point, California, in 2008. This update classifies Group 1 as pulmonary arterial hypertension (PAH) due to idiopathic, heritable, or drug- and toxin-induced causes; it also includes PAH associated with specific disease conditions or persistent pulmonary hypertension of the newborn. Group 1^l^ is PH due to pulmonary veno-occlusive diseases and/or pulmonary capillary haemangiomatosis. Group 2 includes PH due to left heart disease. Group 3 comprises PH due to lung diseases and hypoxia. Group 4 refers to chronic thromboembolic PH. Group 5 encompasses PH due to unclear or multifactorial mechanisms [2].

Pulmonary arterial hypertension though comparatively rare can be very devastating as it progresses rapidly to right heart failure and subsequent occurrence of death within three years of diagnosis. Peak age of incidence is in the 4th and 5th decades of life with a female preponderance. Multiple studies have shown a higher prevalence of SRBD in patients with pulmonary hypertension [1] as well as an increased prevalence of pulmonary hypertension in patients with SRDB (17–53%); and factors such as daytime PO_2_, BMI, and AHI are significantly associated with both [3].

This paper presents a review of the current literature on dysregulatory mechanisms in sleep-related breathing disorders which result in pulmonary hypertension. Emphasis will be placed on Group 3 PH where the broad principles which underlie etiopathogenesis have been elucidated.

## 2. Diagnosis

Accurate diagnosis of PH is based on the acquisition and precise analysis of invasive hemodynamic data as this ultimately determines appropriate treatment options. Current recommendations are for transthoracic echocardiography in the initial screening process with possible subsequent evaluation by right heart catheterization for diagnostic confirmation. Though PH refers broadly to a mean pulmonary artery pressure >25 mmHg from any cause, these invasive studies are crucial to excluding left heart causes of PH where vasodilator therapies should be avoided. For patients with a PCWP <15 mmHg, vasodilator challenge is a crucial diagnostic step for evaluation of vasoreactivity and this is commonly done with inhaled nitric oxide or intravenous agents such as adenosine, epoprostenol, or nitroprusside. All patients with PAH should also undergo routine biochemical, hematologic, immunologic, and thyroid function tests as well as high resolution CT to identify the specific associated condition [2–4].

In view of the association between sleep disordered breathing (SDB) and pulmonary hypertension as depicted by multiple studies [5–17], the American College of Chest Physicians (ACCP) categorizes obstructive sleep apnea (OSA) as a risk factor for PAH. The current ACCP Evidence-Based Clinical Practice Guidelines recommends an assessment for SDB in evaluating patients with PAH and the use of polysomnography when there is clinical suspicion of OSA as the etiology [18, 19].

## 3. Pathophysiology

**Figure 1:**
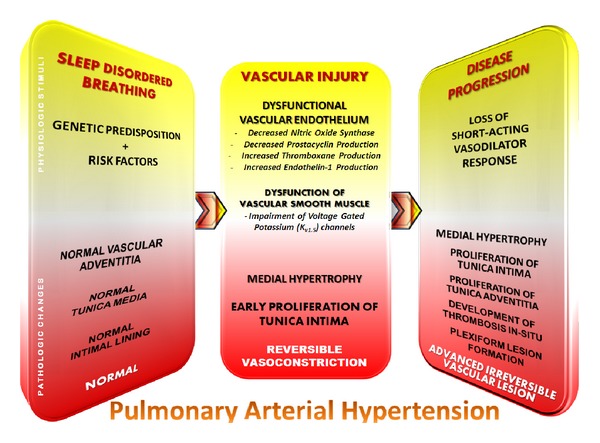
Pathogenesis of pulmonary hypertension [19, 21–23].

The pathological changes observed in PH due to hypoxia and SRBD include medial hypertrophy and obstructive proliferation of the tunica intima within the distal pulmonary arteries. The severity of intimal and medial thickening is highly variable and results in near total occlusion of these vessels. This results in major increments in the pulmonary vascular resistance and considerably impedes blood flow through the lungs. Regions of the lungs with significant emphysematous changes or fibrosis may exhibit substantial destruction of the pulmonary vascular bed. The disordered mechanisms resulting in the observed pathophysiological manifestations are multifactorial (Figure 1). Crucial factors which play a pivotal role in these processes include hypoxic vasoconstriction, mechanical changes resulting from hyper-inflated lungs, capillary loss, and inflammation. New evidence also points to the importance of an imbalance between endothelium-derived factors responsible for vasoconstriction and vasodilation [20].

Compensatory changes occur in the right ventricle to overcome the maladaptive responses of these resistance vessels and improve pulmonary blood flow particularly in situations of increased oxygen demand. Eventually, the right ventricle becomes unable to maintain adequate blood flow and this heralds the onset of dyspnea on exertion. This progresses to overt right ventricular failure and poor cardiac output. Finally, severe debilitation sets in and significant dyspnea occurs at rest; ultimately death occurs in most untreated patients in less than 3 years after initial diagnosis [24, 25]. This drastic clinical course which culminates in significant clinical deterioration of the affected previously healthy individual has resulted in intensified research efforts in search of a definitive cure. Newer treatment modalities have shown moderate improvement in prognosis but fail to halt disease progression or alter eventual mortality data.

## 4. Breathing-Related Sleep Disorders

### 4.1. Normal Sleep

Under healthy conditions cardiovascular regulatory changes occur in specific stages of normal sleep. Nonrapid eye movement (NREM) sleep is associated with a generalized decrease in sympathetic drive; and deeper stages are associated with bradycardia, reduction in blood pressure, stroke volume, cardiac output, vasomotor tone, and systemic vascular resistance. Conversely, REM sleep is characterized by remarkable increases in sympathetic activity and thus labile heart rate and blood pressure values analogous to those observed while in the wakeful state [26]. Dysregulatory cardiovascular changes which characterize sleep-disordered breathing activate neural and circulatory responses with repetitive reflex increases in sympathetic activity. The subsequent vasoconstriction, which ensues, activates mechanisms which result in an eventual rise in mean pulmonary artery pressure [27].

### 4.2. Spectrum of Sleep Disorders

SRBD encompasses several overlapping disorders with varying degrees of severity. These include habitual snoring, increased upper airway resistance syndrome, hypoventilation syndromes, obstructive sleep apnea (OSA), and central sleep apnea (CSA). The most prevalent of the SRBD is OSA which occurs in 4% of all US adult males and 2% of the female population, thus it is the most studied of all SRBD [28]. Poor concentration, fatigue excessive sleepiness, and unrefreshing sleep are some of the characteristic symptoms of these disorders.

Sleep apnea can be defined as repetitive, prolonged airflow cessation with associated sleep arousal and occasional oxygen desaturation. Variants of sleep apnea include obstructive sleep apnea, with persistent respiratory effort in spite of oropharyngeal airway occlusion; central sleep apnea, involving cessation of both airflow and all respiratory effort; and a mixed pattern of both [29]. The term obstructive sleep apnea syndrome is used to refer to the occurrence of obstructive sleep apnea in conjunction with excessive sleepiness and oxygen desaturation. Sleep apnea is commonly characterized by episodes of apnea, hypopnea, intermittent hypercapnia and hypoxia, increased sympathetic activity, and variations in sleep-associated baroreceptor reflex responses. Hypopnea is a reduction in airflow of ≥50% accompanied by an arousal or oxygen desaturation of ≥3%. Respiratory effort-related arousals occur when arousal from sleep result from increasing respiratory effort in the absence of overt apnea or hypopnea; this characterizes increased upper airway resistance syndrome. Change in pulse transit time, nasal pressure measurements, and respiratory inductance plethysmography are common modalities implemented in the assessment of sleep-related respiratory effort. A seemingly “normal” polysomnogram in a symptomatic patient does not rule out the presence of SRBD as the occurrence of respiratory events varies widely in milder variants. A consensus statement by the American Thoracic Society and the American Academy of Sleep Medicine specifies criteria for the diagnosis of SRBD. A diagnosis of obstructive sleep apnea-hypopnea syndrome can only be made in the presence of excessive daytime sleepiness which cannot be better explained otherwise, in the presence of ≥5 obstructed breathing events (including effort-related arousals, apnea, or hypopnea) per hour during sleep (referred to as the respiratory disturbance index (RDI)). Thus, an RDI of 5–15 is mild, 15–30 is moderate; and ≥30 hourly events is classified as severe [30, 31]. 

### 4.3. Obstructive Sleep Apnea

Obstructive sleep apnea is a common chronic SRBD that is characterized by complete or partial airway obstruction with resultant episodes of apnea or hypopnea, respectively. One-fifth of all adults in Western nations have mild OSA, while 1 in 15 adults has moderate to severe OSA. The prevalence is highest in older males with a high body-mass index and features of the metabolic syndrome; yet this disorder is seldom diagnosed and undiagnosed cases are as high as 85% in certain communities. These values are expected to rise in parallel with the current rising trend in obesity worldwide, leading to a resultant increase in the associated cardiovascular comorbidities, depression, and reduced quality of life of affected individuals [32–34].

## 5. Etiopathogenetic Mechanisms

An abrupt withdrawal of the nonchemical respiratory drive accompanies the transition from the wakeful state to NREM sleep resulting in a sudden decline in minute ventilation and pO_2,_ as well as a concurrent rise in pCO_2_ [35]. The decline in sympathetic drive is associated with a reduction in heart rate and cardiac output; normal individuals in NREM sleep experience a decline of up to 20% in systemic blood pressure [36]. In contrast, parasympathetic tone and pulmonary artery pressure rise during sleep [37]. In REM sleep however, the respiratory drive is influenced by behavioral changes and inhibition of the resting muscle tone in the upper airway musculature and accessory muscles, resulting in irregular breathing patterns which may worsen the hypoxemia and hypercapnia. About 80% of sleep time is spent in the NREM phase while 20% is in REM [28].

### 5.1. Changes in Cardiovascular Physiology in Sleep-Disordered Breathing

Recent studies have shown that individuals with OSA are at significant risk of developing systemic hypertension, cerebrovascular events, and ischemic heart disease. These result from acute cardiovascular changes which gradually become chronic and lead to cardiac remodeling and altered cardiovascular hemodynamics [38, 39]. Key factors influencing these alterations include an abnormal amplification of negative intrathoracic pressure in the presence of a closed glottis as well as hypoxemic episodes and sleep arousals. These changes fluctuate acutely between episodes of apnea and ventilation, with variable chronotropic and vasomotor responses among individuals, and ultimately result in autonomic dysfunction, hypercoagulability, and a predisposition to thromboembolic events [21, 40–42].

### 5.2. Acute and Transient Cardiovascular Effects

**Figure 2:**
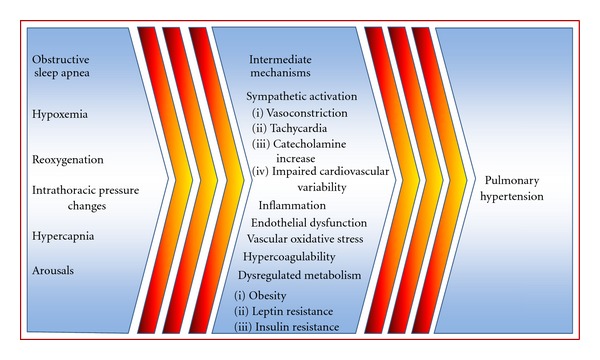
Intermediate mechanisms which potentially increase the risk of developing pulmonary hypertension in obstructive sleep apnea. These intermediate mechanisms in obstructive sleep apnea may contribute to initiating and perpetuating pathologic cardiovascular changes which result in pulmonary hypertension.

A physiologic reduction in systemic blood pressure of up to 15% occurs during stages 3 and 4 of NREM sleep accompanied by a 10% reduction in cardiac output; this results in an overall decline in systemic vascular resistance. More complex hemodynamic responses occur in response to apneic stimuli which causes pulmonary and systemic hypertension, increased afterload, and reduction in cardiac output (Figure 2). These alterations to normal physiology are a consequence of changes in intrathoracic pressure, sympathetic activation, and episodes of hypoxia and hypercapnia [43, 44].

#### 5.2.1. Negative Intrathoracic Pressure

A hallmark of sleep apnea is the Mueller maneuver (inspiration against a closed upper airway) which could generate negative intrathoracic pressures with values as low as −80 cm H_2_O. The altered cardiac configuration and chamber filling pressures may consequently increase left ventricular transmural pressure and afterload, while LV relaxation is impaired by the exaggerated negative intrathoracic pressure thus worsening LV filling. This reduces stroke volume and cardiac output while the negative intrathoracic pressure stretches the aortic wall and activates intramural baroreceptors with episodic inhibition of sympathetic outflow with each Mueller maneuver. Increased venous return which occurs as the individual resumes breathing causes right ventricular distention and a leftward interventricular septal shift (ventricular interdependence) compromising LV diastolic filling and compliance [45–51]. 

#### 5.2.2. Sympathetic Activation

Transient rises in sympathetic activity with vasoconstriction and hypertension accompany episodes of apnea with the lowest blood pressure values recorded at the midpoint of these episodes. Blood pressure then rises gradually with a sudden elevation at onset of breathing. Apneic episodes that exceed 35 seconds are characterized by decline in cardiac output of about 33%; however cardiac output rises by up to 15% above baseline at resumption of breathing [52–55]. Elevated systemic blood pressure and reduced cardiac output indicate apnea-related increase in systemic vascular resistance, with alpha-sympathetic neurons mediating vasoconstriction. Pulmonary artery pressures are noted to rise acutely in hypoxia and at onset of breathing in conjunction with systemic blood pressure; these neuronal effects are primarily in response to hypoxemia and hypercapnia [56–59]. With prolonged apnea and increasing hypoxemia, bradycardia worsens. Though tachy and bradyarrhythmias, sinus pauses, ventricular ectopy, and complete heart block are frequently observed in patients with obstructive sleep apnea syndrome, ventricular arrhythmias become more frequent at significant hypoxemia [60–64].

#### 5.2.3. Effects of Hypoxia

Activation of carotid chemoreceptors by hypoxemia triggers arteriolar vasoconstriction and systemic catecholamine secretion. This response is most marked in the systemic vascular bed at oxyhemoglobin saturation levels lower than 65% and leads to transient hypertension [65].

Conversely, pulmonary vasoconstriction is a direct response to alveolar hypoxia in a physiologic attempt to minimize ventilation perfusion mismatch. The recurrence of hypoxemic episodes in sleep apnea result in repetitive increases in pulmonary artery pressures; however, about 1 in 5 patients develops sustained pulmonary hypertension during the daytime [66–70]. More severe OSA and hypoxia may lead to right ventricular hypertrophy culminating in daytime pulmonary hypertension and right ventricular failure in the presence of hypercapnia and chronic alveolar hypoventilation [71–74].

### 5.3. Mechanisms Linking Obstructive Sleep Apnea to Chronic Cardiovascular Disease

#### 5.3.1. Oxidative Stress

Repetitive bouts of nocturnal hypoxemia and intermittent reperfusion which accompany apneic episodes may generate highly reactive superoxide radicals as well as reperfusion-mediated endothelial damage, thus increasing susceptibility to atherosclerosis. Several polymorphonuclear leukocytes respond to hypoxemia with the release of free oxygen radicals; the cumulative effect of repetitive cycles of hypoxia followed by reoxygenation occurring multiple times in each hour of sleep over decades in patients who remain untreated may further worsen this preexisting vascular oxidative stress. The use of CPAP in patients with sleep apnea has been shown to reduce superoxide production [73–79].

#### 5.3.2. Sympathetic Activation

A high level of sympathetic tone has been observed in patients with sleep apnea and administration of 100% oxygen results in deactivation of the chemoreceptor reflex response and significant reduction in sympathetic activity [80–82]. This has also been linked to increased resting heart rate and blood pressure variability and a reduced heart rate variability which all increase cardiovascular risk. The decrease in heart rate variability has been associated with an increase in cardiovascular mortality [83–87].

#### 5.3.3. Vascular Endothelial Dysfunction

The release of vasoactive substances and vascular endothelial dysfunction may follow recurrent bouts of hypercapnia, hypoxia, and changes in vasomotor tone. Surges in plasma endothelin levels may help sustain vasoconstriction and endothelial dysfunction as observed in hypertension, dyslipidemia, and smoking, and diabetes has also been demonstrated in persons with OSA in the absence of other overt cardiovascular co-morbidity [88–90].

#### 5.3.4. Metabolic Dysregulation

Dysregulation of metabolic pathways associated with OSA may heighten cardiovascular risk and increase the predilection for weight gain. Leptin, a hormone derived from adipocytes and primarily responsible for appetite suppression, demonstrates increased levels in obesity possibly from resistance to its metabolic effects. This hormone, which has been identified as an independent cardiovascular risk marker and might induce platelet aggregation, has been observed to occur at much higher levels in OSA than in obesity. Moreover, patients with OSA have been observed to develop significant weight gain in the year immediately preceding diagnosis, and treatment of OSA with CPAP decreases leptin levels and accumulation of visceral fat, further implicating leptin resistance in the predisposition to weight gain [91–97].

Impaired glucose tolerance may also result from OSA. Elevated fasting blood glucose, serum insulin, and HbA_1c_ have been observed in these individuals with a direct correlation between severity of insulin resistance and OSA; patients with severe OSA are five times more likely to develop overt diabetes mellitus than the general population [22, 98–101].

#### 5.3.5. Inflammation

Serum levels of C-reactive protein (CRP) and inflammatory cytokines such as interleukin-6 and tumor necrosis factor-*α* may be increased in response to hypoxia and sleep deprivation, both of which are present to varying degrees in patients with OSA. Patients with OSA have also shown elevated levels of these cytokines. The inhibition of nitric oxide synthase mediated by CRP which also increases the expression of certain cell adhesion molecules may worsen endothelial dysfunction and further aggravate preexisting vascular disease [102–111]. The expression of cell adhesion molecules which mediate leukocyte adhesion to endothelial cells may be directly modulated by hypoxic stress, thus leading to elevated levels of cell adhesion molecules in persons with moderate to severe OSA. This elevation may be reversed with the use of CPAP therapy.

#### 5.3.6. Coagulation

Increased nocturnal catecholamine levels in conjunction with other factors increase the tendency for platelet aggregation in OSA; a condition almost completely reversed by the use of CPAP. Similarly, an increase in fibrinogen level, hematocrit and hyperviscosity of blood result in a predilection for thromboembolism and atherosclerosis. The reversal of these hypercoagulable phenomena with CPAP therapy suggests a causal relationship to OSA [23, 112–118].

## 6. Right Heart Dysfunction and Pulmonary Hypertension in Sleep-Disordered Breathing

OSA is frequently regarded as an independent risk factor in the development of pulmonary hypertension and subsequent cor pulmonale. However, studies show a stronger association between PH and obstructive ventilatory patterns observed on pulmonary function testing as well as daytime hypercapnia and hypoxemia; where most of this association is attributed to coexisting obstructive airway disease. PH correlates highly with elevated waking pCO_2_, reduced waking pO_2_, coexisting obstructive pulmonary disease, and body mass index, particularly in severe cases [119–121]. Patients with pulmonary hypertension have also been shown to have more lengthy periods of hypoxemia. The high correlation of PH with increased BMI, reduced vital capacity, expiratory reserve volume, and total lung capacity suggest that the association between PH and OSA is strongest in the presence of the mechanical consequences of obesity on respiration [8, 14, 123]. Recurrent and persistent pressure and volume strains on the right heart increase wall tension in the right ventricle facilitating myocardial hypertrophy. Chronic hypoxemia resulting from episodic nocturnal oxygen desaturations potentiates the development of permanent PH by the induction of vascular remodeling [67, 125, 127]. 

## 7. Treatment of Pulmonary Hypertension in Sleep-Related Breathing Disorders

Pulmonary vascular response to hypoxia has been shown to reduce with significant drop in the mean pulmonary artery pressures after the therapeutic use of nasal CPAP, suggesting potential reversibility of pulmonary hypertension upon treatment of OSA [128–130]. Other therapeutic strategies implemented in recent times include the use of hemodialysis in patients with coexisting chronic renal failure to reduce the severity of OSA [131]. Surgical alternatives and cardiac atrial pacing have also been explored as therapeutic alternatives [132]; in patients with less tolerance for conventional treatment strategies, the use of agents that limit effects of inflammatory mediators such as aspirin or statins may be beneficial [130, 134].

## 8. Summary

SRBD encompasses conditions which range from habitual snoring to obstructive sleep apnea and may be associated with considerable morbidity and mortality. Increasing evidence points to the significant association between SRBD and PH. Though the majority of research endeavors in recent times have focused on left heart hemodynamics, few studies have attempted to outline the effects of SRBD on the pulmonary vascular system and multiple studies are ongoing to further elucidate the specific pathways underlying these mechanisms. Available evidence indicates that the development of pulmonary hypertension in patients with SRBD involves the complex interplay of multiple factors and correlates strongly with the severity and duration of nocturnal desaturations as well as associated risk factors. Early recognition and treatment may effectively reduce these complications.
